# The Reproductive Outcome of Women with Hypogonadotropic Hypogonadism in IVF

**DOI:** 10.3389/fendo.2022.850126

**Published:** 2022-06-06

**Authors:** Chun-mei Zhang, Hua Zhang, Rui Yang, Li-xue Chen, Ping Liu, Rong Li, Jie Qiao, Ying Wang

**Affiliations:** ^1^ Center for Reproductive Medicine, Department of Obstetrics and Gynecology, Peking University Third Hospital, Beijing, China; ^2^ National Clinical Research Center for Obstetrics and Gynecology, Peking University Third Hospital, Beijing, China; ^3^ Key Laboratory of Assisted Reproduction, Ministry of Education, Peking University, Beijing, China; ^4^ Beijing Key Laboratory of Reproductive Endocrinology and Assisted Reproductive Technology (Peking University Third Hospital), Beijing, China; ^5^ Research Center of Clinical Epidemiology, Peking University Third Hospital, Beijing, China

**Keywords:** hypogonadotropic hypogonadism, *in vitro* fertilization and embryo transfer, infertility, cumulative live birth rate, anovulation

## Abstract

**Objective:**

The purpose of this study was to evaluate the reproductive outcome of patients with hypogonadotropic hypogonadism (HH) receiving *in vitro* fertilization and embryo transfer (IVF-ET).

**Methods:**

The reproductive outcome of 81 HH patients and 112 controls who underwent oocyte retrieval was evaluated retrospectively in the Center for Reproductive Medicine of Peking University Third Hospital from 2010 to 2019.

**Results:**

The basic levels of follicle stimulating hormone (FSH), luteinizing hormone (LH), estradiol (E2), androstenedione (A) and prolactin (PRL) were significantly lower in the HH group than the control group. Although the HH patients required a significantly longer stimulation and higher gonadotropin (Gn) doses than the control patients, the total number of oocytes retrieved, fertilized embryos, two pronuclear (2PN) embryos, transferable embryos, fertilization and 2PN rates were comparable between the two groups. Although the live birth rate (LBR) of the first fresh cycle was higher in the control group than the HH group, there was no statistical significance. Then we further divided HH patients into two subgroups according to the etiology. Forty-one cases were termed as congenital HH (CHH), while the other 40 cases were termed as acquired HH (AHH), the latter includes functional hypothalamic amenorrhea (FHA) and pituitary HH (PHH). Our results showed that there were no significant differences in basic clinical characteristics and IVF parameters between the two groups. In the HH group, a total of 119 oocyte retrieval cycles were carried out and they responded adequately to ovulation induction. Urinary human menopausal gonadotropin (HMG) was used alone in 90 cycles while combination of HMG and recombinant human follicle stimulating hormone (rFSH) in the other 29 cycles. There were no significant differences in IVF-related parameters between the two groups. The conservative cumulative live birth rates (CLBRs) after the first, the second and ≥third cycles were 43.21%, 58.02% and 60.49%, respectively, while the corresponding optimal CLBRs were 43.21%, 68.45% and 74.19%. The preterm birth (PTB) rates of singletons and twin pregnancy in HH patients were 8.33% (3/36) and 30.77% (4/13), respectively.

**Conclusion:**

IVF-ET is an effective treatment for HH patients with infertility and patients can get satisfactory pregnancy outcomes.

## Introduction

Hypogonadotropic hypogonadism (HH) is caused by deficiencies in hypothalamic endogenous gonadotropin-releasing hormone (GnRH) release or pituitary gonadotropin (Gn) secretion, leading to an imbalance in the hypothalamic-pituitary-gonadal (HPG) axis and diminished ovarian function. From a reproductive health perspective, HH is classified as WHO type I ovulatory disorder and the diagnosis of HH should differentiate from other forms of primary or secondary amenorrhea. The basic approach is an assessment of the sex hormones, and other diagnostic tools include but are not restricted to pelvic examination, abdominal or transvaginal ultrasound, progestin challenge, cerebral magnetic resonance imaging (MRI) scan, detailed personal history collection with a focus on diet, eating disorders, exercise, weight, development, menstruation and mental distress, bone mineral density test, detection of thyroid hormones, prolactin (PRL) determination, and GnRH stimulation test. The causes of HH can be congenital or acquired, and according to the pathophysiology, HH is categorized into congenital HH (CHH), pituitary HH (PHH), and functional hypothalamic amenorrhea (FHA). CHH is caused by deficient production, secretion or action of GnRH, and characterized by incomplete or absent puberty and infertility, which can present solely as congenital GnRH deficiency or be associated with other developmental anomalies (1). When associated with anosmia or hyposmia, CHH is termed as Kallmann syndrome, which results from abnormal embryonic migration of GnRH neurons from their origin in the olfactory placode to the forebrain (2). The diagnosis of CHH is necessarily one of exclusion, since it requires acquired, functional or structural conditions to be ruled out. CHH is genetically a heterogeneous disorder with identified X-linked, autosomal dominant, and autosomal recessive patterns of inheritance. With the development of next-generation sequencing (NGS) techniques, pathogenic variants in more than 50 genes have been identified in CHH (3). However, each gene linked to CHH only seems to underpin a small percentage of total patients, so we are still far from achieving a comprehensive understanding of the genetic basis of CHH. Patients have generally not benefited from advances in genetics in respect of novel therapies (4). FHA is a form of chronic anovulation that is not due to identifiable organic causes. Several etiologic factors have been well described for FHA, including intense or frequent exercise, weight loss, psychological stress, and psychological disturbance (5, 6), while in many, no eliciting factors can be identified. Importantly, even in those for whom causative factors have been suggested, the precise mechanism of disruption of GnRH secretion has not been elucidated. Previous reports identified several rare genetic variants in genes related to GnRH deficiency in FHA patients. Therefore, patients with FHA may have a genetic basis partially in common with idiopathic CHH and the total load of mutations in genes related to GnRH action might be less in FHA than that in idiopathic CHH (7). Common causes related with PHH includes infiltrative or infectious pituitary lesions, pituitary apoplexy, and radiation.

Due to the long-term deficiency stimulation of Gn, women with HH show symptoms of hypoestrogenism, hypoplasia of uterus, ovulation inefficiency, and amenorrhea. Of note, the management of HH with each category requires different approaches. The principal target of treatment for CHH is supplying exogenous Gn or sex steroid hormones depending on the timing and aim of treatment whether it is pubertal induction, general health or fertility (8). For FHA patients, we need to find the predisposing factors, provide psychological support and improve energy balance. In addition to the above management, the management of women with PHH is distinctive from CHH because they require extensive multidisciplinary input with endocrinologists to address the cause and the different pituitary functions affected (9). Due to estrogen deficiency, the health in HH patients is disturbed in several aspects including their skeletal system, cardiovascular system and mental problems (10). Herein we should also pay attention to its long-term complications.

Anovulation and infertility are common complaints among women with HH who require hormonal therapeutic intervention. For patients with fertility requirements, artificial hormone replacement (HRT) with estrogen and progesterone are primarily applied to promote uterus development. Then, when the size of uterus is normal, ovarian stimulation with Gn or pulsatile GnRH is used to induce ovulation. Pulsatile GnRH treatment has been utilized for the management of women with hypothalamic HH suffering from infertility. However, pulsatile GnRH requires near perfect compliance and close monitoring. In addition, the use of a portable pump injection device and the need to inject subcutaneously or intramuscularly has been regarded as a disadvantage (11). In clinical practice, the more commonly used ovulation induction procedure for patients with HH is daily low-dose injections of Gn, which is applicable to all types of HH. HH women do not have endogenous luteinizing hormone (LH), therefore, Gn with an LH component is required. The development of urinary-derived Gn (human menopausal gonadotropin, HMG) containing both follicle stimulating hormone (FSH) and LH a few decades ago paved the way to replacing the absent endogenous hormones. The prognosis for inducing ovulation with HMG in most of the HH patients is favorable through guided coitus or intrauterine insemination (IUI). However, a few of patients may present with ovulation induction failure or multiple ovarian follicles development leading to ovarian hyperstimulation syndrome (OHSS). Besides, some other couples are unable to be pregnant naturally due to fallopian tube, male factors or other causes.

Assisted reproductive technology (ART), including *in vitro* fertilization (IVF), has developed in recent years and is a better effective choice for the patients who fail to be pregnant through ovulation induction. However, the low incidence of HH makes it difficult to evaluate the reproductive outcome of IVF in women with HH. Limited studies have shown that, all categories of HH are considered as a whole group to compare IVF characteristics with control group (12–14). However, whether there are differences in IVF characteristics among patients with different types of HH have not been reported. Due to the long-term deficiency of endogenous estrogen stimulation, how HH patients respond to exogenous Gn and their pregnancy outcomes especially the live birth rate (LBR) are still unknown. Besides, since the uterine development may be impaired after long-term amenorrhea, whether HH patients have increased pregnancy risks, such as late pregnancy abortion and preterm labor, is also unclear. In this study, we report a retrospective analysis performed on 81 patients with HH and 112 controls in the Center for Reproductive Medicine of Peking University Third Hospital with the aim to analyze the clinical IVF outcomes of women with HH. Furthermore, we also made a detailed analysis on IVF outcomes among patients with different types of HH.

## Materials and Methods

### Patients

A total of 81 women with HH who underwent oocyte retrieval in the Reproductive Center of Peking University Third Hospital from 2010 to 2019 were included. The diagnosis of HH was based on the primary or secondary amenorrhea, absence of withdrawal bleeding following a progestin challenge test, and normal or low levels of serum FSH and LH (13). Other causes of amenorrhea, such as polycystic ovary syndrome, uterine disorder or ovarian dysfunction were excluded. Combined with the patient’s medical history and auxiliary examination results, 41 patients were classified as CHH group, 31 patients as FHA group and the other 9 patients as PHH group. Here, due to the small number of patients with PHH, we grouped them with FHA and termed as acquired HH (AHH). Most patients experienced previous multiple ovulation induction with or without IUI or IVF cycles and failed to get pregnancy, and some other patients received IVF because of oviduct factors. The major cause for IVF was the failure to be pregnant after guided coitus or IUI with ovulation induction. Fourteen of them underwent intracytoplasmic sperm injection (ICSI) for coexisting male factor infertility. As a control group who underwent IVF with matched background characteristics during the same period, 112 women diagnosed with tubal factor or male factor infertility were also evaluated.

### Controlled Ovarian Stimulation Protocols

#### Ovulation Induction Protocol for HH Patients

At least 3 cycles of HRT were prescribed to promote uterus development before initiating the COS on the second or third day of menstruation. Then they underwent a total of 119 oocyte retrieval cycles. Urinary gonadotropin for injection (HMG, Livzon, China) was used alone in 90 cycles while combination of HMG and recombinant human FSH for injection (rFSH, Gonal-F, Merck Serono, Germany) in the other 29 cycles. Serum levels of estradiol (E2), progesterone (P), FSH, and LH were detected on the second day of menstruation and the monitoring was performed using ultrasound, and then Gn dosage was adjusted based on the individual patient’s ovarian response.

#### Ovulation Induction Protocol for Control Patients

In the control group, GnRH agonist (GnRH-a) or antagonist (GnRH-ant) regimes were used. In the long or ultra-long GnRH-a protocol, Triptorelin Acetate (Diphereline, Ipsen, France) with a dose of 1.8 mg or 3.75 mg was administered in the mid-luteal phase or the menstrual period of the previous menstrual cycle. Fourteen or 28 days later, rFSH was administered after pituitary suppression was demonstrated. In GnRH-ant regime, rFSH was administered on the second day of menstruation and pituitary suppression was managed with Cetrorelix (Cetrotide, Merck Serono, Germany) starting on the sixth or seventh day of the cycle according to the follicular growth (when the leading follicle reached a diameter of 13-14 mm).

For all the subjects, 10000 IU human chorionic gonadotropin (HCG, Livzon, China) or 250 ug recombinant human choriogonadotropin alfa solution for injection (rHCG, Ovidral, Merck Serono, Germany) were triggered when at least the diameter of 3 dominant follicles reached 18 mm and oocyte retrieval was usually performed with transvaginal ultrasound 36 h after HCG injection. Conventional IVF or ICSI was performed depending on the semen analysis on the day. Two embryos or one blastocyst transfer was performed on the day of 3 or 5 later after oocyte retrieval. Serum β-HCG was measured 14 days following embryo or blastocyst transfer. Clinical pregnancy was defined as a positive pregnancy blood test followed by the presence of gestational sac on transvaginal ultrasound 30 days after the embryo or blastocyst transfer. Luteal support was maintained until 10 weeks of gestation if conception occurred.

### Statistical Analysis

R4.0.3 statistical software was used for statistical analysis. The quantitative data were given as means ± standard deviations or as medians ± quartile (25% quantile, 75% quantile), as appropriate. Qualitative data were expressed by count and percentage. Age was compared between the two groups using an independent sample T test. Wilcox test was employed to analyze the differences of IVF parameters between HMG and combination groups. Chi-square test was used to compare the rates or proportions. A *P* value of less than 0.05 (2-sided significance testing) was considered to be statistically significant.

## Results

### Clinical Characteristics and IVF Outcomes of Patients

The characteristics of the 81 women with HH and 112 controls are shown in **Table 1**. There were no differences in age or body mass index (BMI) between the HH group and the control group. But the basic levels of FSH, LH, E2, androstenedione (A), and PRL were significantly lower in the HH group than those in the control group.

**Table 1:** Comparison of clinical characteristics between hypogonadotropic hypogonadism (HH) and control groups.

Variables	HH group (n=81)	Control (n=112)	*P*
Age (y)	30 (27, 32)	30 (29, 32)	0.669
BMI (kg/m^2^)	21.01 (19.53, 23.60)	21.49 (19.58, 23.65)	0.578
Basal serum hormonal level			
FSH (mIU/mL)	1 (0.36, 2.88)	6.71 (5.43, 8)	< 0.001
LH (mIU/mL)	0.3 (0.1, 0.78)	3.36 (2.37, 4.73)	< 0.001
E2 (pmol/L)	82.2 (73.4, 119)	157.42 (121.25, 221.38)	< 0.001
A (nmol/L)	4.54 (3.21, 6.06)	6.3 (4.8, 8.38)	< 0.001
PRL (ng/mL)	5.65 (4.28, 8.68)	12 (8.91, 17.7)	< 0.001

BMI, body mass index; FSH, follicle stimulating hormone; LH, luteinizing hormone; E2, estradiol; A, androstenedione; PRL, prolactin. The data are expressed by the median (25% quantile, 75% quantile), and the comparison between the two groups is performed by Wilcox test.

We have compared the IVF characteristics of the first fresh cycle of HH patients with control group. And the outcomes are detailed in **Table 2**. The HH patients required a significantly longer stimulation and higher Gn doses than the control patients. Although larger amounts of Gn were used, the serum LH and P levels on HCG day were still significantly lower in HH patients than that in control patients. E2 level was also higher in the control group, but the difference was not statistically significant. The total number of oocytes retrieved, fertilized embryos, two pronuclear (2PN) embryos, transferable embryos, fertilization rate, and 2PN rate were comparable between the two groups. In the HH groups, 63 cases received fresh cycles transplantation and 23 cases got a live birth, while in the control group, 42 cases delivered among the 88 patients who underwent their fresh transplantation cycle. Although the live birth rate (LBR) of the fresh cycle was higher in the control group than that in the HH group, there was no statistical significance. In our study, GnRH-a regime was used in 59 patients and GnRH-ant regimes was used in 53 patients. Then we further compared HH group with the two regimes respectively and found that the total number of oocytes retrieved, fertilized embryos and 2PN embryos were higher in the GnRH-a regime group than that in the HH patients, but the number of transferable embryos, fertilization and 2PN rate were comparable between the two groups, while compared with the GnRH-ant regime group, no differences in IVF outcomes were observed. Moreover, a larger amount of Gn and a longer duration of ovarian stimulation were necessary in HH patients regardless of whether GnRH-a and GnRH-ant regimes were selected in the control group (**Supplementary Tables 1** **,** **2**).

**Table 2:** Comparison of cycle characteristics between hypogonadotropic hypogonadism (HH) and control groups.

Variables	HH group (n=81)	Control (n=112)	*P*
Hormone levels on HCG day			
E2 (pmol/L)	6751 (4524.5, 12656)	10251 (5678.75, 13948.75)	0.128
LH (mIU/mL)	0.18 (0.11, 0.56)	1.01 (0.6, 2.32)	< 0.001
P (nmol/L)	1.77 (1.42, 3.09)	2.45 (1.56, 3.57)	0.035
Duration of stimulation (d)	14 (13, 16)	11 (10, 12)	< 0.001
Total amount of Gn injected (IU)	3487.5 (2850, 4500)	2550 (1715.62, 3318.75)	< 0.001
No. of oocytes retrieved	11 (7, 14)	11.5 (8, 17)	0.177
No. of fertilized embryos	9 (5, 11)	8 (5, 12.25)	0.512
No. of 2PN embryos	6.5 (4, 9)	7 (4, 10)	0.334
No. of non-2PN embryos	1 (0, 2)	1 (0, 2)	0.464
Fertilization rate (%)	0.79 (0.64, 0.97)	0.76 (0.62, 0.89)	0.244
2PN rate (%)	0.86 (0.72, 1)	0.86 (0.73, 1)	0.497
No. of transferable embryos	3.5 (2, 8)	4 (2, 8.25)	0.704
LBR (%)	36.5 (23/63)	47.7 (42/88)	0.170

HCG, human chorionic gonadotropin; E2, estradiol; LH, luteinizing hormone; P, progesterone; Gn, gonadotropin; PN, pronuclear; LBR, live birth rate. The data are expressed by the median (25% quantile, 75% quantile), and the comparison between the two groups is performed by Wilcox test and Pearson Chi square test.

### IVF Outcomes

Then we focused on pregnancy outcomes in patients with HH. A total of 119 oocyte retrieval cycles were carried out in the 81 patients. Among them, 27 patients underwent 2 while another 10 patients underwent 3 oocyte retrieval cycles. Only 1 patient underwent 4 cycles. All the HH patients responded adequately to ovulation induction. Transferable embryos were obtained in 112 cycles, accounting for 94.12%. Only 1 cycle had no oocyte and another 6 had no embryos to transfer after the oocyte retrieval. Embryos were transferred in 92 fresh cycles and frozen in 20 cycles. This decision of embryo frozen was made for 13 cases to prevent the risk of OHSS and no severe OHSS was detected in all patients, and the other 7 patients for elevation of P or personal reasons. According to the medicine for ovulation induction we used, the 119 cycles were divided into two groups, one group used HMG (90 cycles) alone while another group used HMG combined with rFSH (29 cycles). IVF outcomes were compared between the two groups and there were no significant differences in IVF-related parameters between HMG and HMG combined with rFSH groups (**Table 3**).

**Table 3:** Comparison of IVF related parameters between HMG and combined medication group.

Variables	HMG (n=90)	Combination group (n=29)	*P*
Hormone levels on HCG day			
E2 (pmol/L)	7114.5 (5072.5, 11454.25)	6894 (3744, 14798)	0.56
LH (mIU/mL)	0.18 (0.11, 0.57)	0.18 (0.11, 0.36)	0.65
P (nmol/L)	1.75 (1.21, 3.06)	2.25 (1.44, 3.28)	0.54
Duration of stimulation (d)	14 (13, 16)	14 (12, 15)	0.45
Total amount of Gn injected (IU)	3825 (2962.5, 4762.5)	3975 (3075, 4625)	0.96
No. of oocytes retrieved	10 (7.25, 14)	12 (7,15)	0.64
No. of fertilized embryos	8 (6, 10)	7 (5, 11)	0.47
No. of 2PN embryos	6 (5, 9)	5 (3, 9)	0.30
No. of non-2PN embryos	1 (0, 2)	2 (1, 2)	0.35
Fertilization rate (%)	0.81 (0.67, 0.93)	0.72 (0.52, 0.89)	0.16
2PN rate (%)	0.86 (0.71, 1)	0.8 (0.67, 0.94)	0.29
No. of transferable embryos	3 (2,7)	3 (2,6)	0.59

HCG, human chorionic gonadotropin; E2, estradiol; LH, luteinizing hormone; P, progesterone; Gn, gonadotropin; PN, pronuclear. The data are expressed by the median (25% quantile, 75% quantile), and the comparison between the two groups is performed by Wilcox test.

Cumulative live birth rate (CLBR), which represents the total chance rate of LBRs of each retrieval cycle after all the embryos obtained are transferred, was calculated (15). Conservative CLBR assumed that women who did not return for treatment would not have a live birth, whereas optimal CLBR assumed that these women would have LBRs similar to those for women continuing treatmentcs. In the first cycle of 81 patients, 35 cases were successful in pregnancy and delivery, and the LBR was 43.21%. Twenty-seven cases underwent the second cycle of oocyte retrieval, 12 cases delivered and the LBR was 44.44%. The third cycle for oocyte retrieval was performed in 10 patients, but only one succeed. We had one patient performing the fourth cycle and finally got a live birth. As the number of patients having three or more treatment cycles was small, patients who completed more than three cycles were grouped into one group for analysis. The conservative CLBRs after the first, the second, and ≥ third cycles were 43.21%, 58.02%, and 60.49%, respectively, while the corresponding optimal CLBRs were 43.21%, 68.45%, and 74.19% (**Table 4**).

**Table 4:** The cumulative live birth rates (CLBRs) over multiple complete IVF cycles of HH patients.

Cycle number	1^st^	2^nd^	≥3^rd^
No. of cycles (n)	81	27	11
No. of patients with at least one live birth (n)	35	12	2
LBR per cycle (n/N%)	43.21	44.44	18.18
Conservative CLBR (%) (95%CI)	43.21 (32.42, 54.00)	58.02 (47.28, 68.77)	60.49 (49.85, 71.14)
Optimal CLBR (%) (95%CI)	43.21 (32.42, 54.00)	68.45 (58.33, 78.57)	74.19 (64.66, 83.72)

LBR, live birth rate.

The definition of preterm birth (PTB) is the delivery prior to 37 weeks. Among the 49 live births, there were 36 singletons, including 33 full-term births, 3 PTBs and 13 twins, including 9 full-term births and 4 PTBs. Only 1 preterm infant delivered at 29 weeks of gestation, and the other 6 delivered at 36 weeks.

Another interesting question was that whether there was a difference in IVF outcomes between patients with different HH categories. Our results showed that there were no significant differences in basic clinical characteristics and IVF parameters between CHH and AHH groups (**Table 5**). Besides, we also made a comparative analysis between CHH and FHA groups, and no significant differences were detected (**Supplementary Table 3**).

**Table 5:** Comparison of clinical and cycle characteristics between congenital hypogonadotropic hypogonadism (CHH) and acquired hypogonadotropic hypogonadism (AHH) groups.

Variables	CHH (n=41)	AHH (n=40)	*P*
Age (y)	29 (27, 32)	31 (28.75, 32.5)	0.090
BMI (kg/m^2^)	21.48 (19.48, 24.24)	20.97 (20.1, 23.59)	0.706
Basal serum hormonal level			
FSH (mIU/mL)	0.83 (0.28, 2.98)	1.13 (0.4, 2.55)	0.775
LH (mIU/mL)	0.27 (0.1, 1.02)	0.38 (0.1, 0.75)	0.844
E2 (pmol/L)	91.4 (73.4, 139)	73.4 (73.4, 107.5)	0.094
A (nmol/L)	4.7 (3.77, 6.2)	4.06 (2.98, 5.86)	0.322
PRL (ng/mL)	5.6 (4.58, 6.9)	6.31 (3.79, 9.91)	0.762
Hormone levels on HCG day			
E2 (pmol/L)	6575 (4302, 10984)	6960 (5611, 13117.5)	0.643
LH (mIU/mL)	0.18 (0.11, 0.58)	0.18 (0.11, 0.41)	0.434
P (nmol/L)	1.69 (1.34, 3.4)	1.84 (1.4, 2.61)	0.640
Duration of stimulation (d)	14 (13, 17)	14 (13, 15)	0.375
Total amount of Gn injected (IU)	3537.5 (3018.75, 4631.25)	3337.5 (2568.75, 4218.75)	0.204
No. of oocytes retrieved	11 (7, 12)	12 (7, 16)	0.146
No. of fertilized embryos	8 (5, 9)	9 (6, 12.25)	0.127
No. of 2PN embryos	6.5 (3, 9)	6.5 (5, 9.5)	0.252
No. of non-2PN embryos	1 (0, 2)	2 (0.75, 2)	0.358
Fertilization rate	0.77 (0.63, 0.94)	0.81 (0.66, 0.97)	0.716
2PN rate	0.85 (0.67, 1)	0.85 (0.75, 0.96)	0.547
No. of transferable embryos	3 (2, 8.25)	4 (2, 8)	0.223

BMI, body mass index; FSH, follicle stimulating hormone; LH, luteinizing hormone; E2, estradiol; A, androstenedione; PRL, prolactin; HCG, human chorionic gonadotropin; Gn, gonadotropin; PN, pronuclear. The data are expressed by the median (25% quantile, 75% quantile), and the comparison between the two groups is performed by Wilcox test.

## Discussion

HH is one of the least common etiologies for female infertility and there are only a few studies on IVF characteristics in this rare condition. The key to the success of IVF is to obtain sufficient and high-quality oocytes and embryos, which is closely related to the dosage of Gn used in IVF. Generally, one patient’s age and ovarian reserve function are the mainly considerations when we determine the Gn dosage of ovulation induction. However, it is unlikely that standard measures of the remaining oocyte pool (baseline FSH and LH levels or antral follicle numbers) will be of use, as they cannot be accurately interpreted in this population of women who have low Gn levels and lack cyclical menses. Besides, the age-dependent decline in ovarian response for patients with HH has not been established. In these situations, the question arises at which dose to start stimulation. In our study, 96 cycles started the ovarian stimulation with Gn dose between 150-300 IU, accounting for 80.67%. Although patients with HH have a long-term estrogen deficiency, their response to COS treatment was similar to the control women. 94.12% cycles obtained transferable embryos and only 7 cycles had no oocytes or embryos for transfer. The effect of ovulation induction was satisfactory and no severe OHSS occurred, providing a dose reference for clinical practice in the future.

Previous studies have documented that a larger amount of Gn and a longer duration of ovarian stimulation are needed in HH patients than that in tubal factor patients (12, 14), explained by the “dormant” ovaries that need to be primed before follicular response is achieved. However, the mean number of retrieved oocytes, implantation, fertilization, and pregnancy rates are not significantly different in comparison to the tubal group patients (12, 16). Ghaffari et al. found that despite a higher fertilization rate and higher number of grade A/B embryos transferred in the tubal factor group, the implantation, pregnancy, and LBRs are similar between HH groups and tubal factor group (13). In consistent with previous findings, our study confirmed that HH patients required a significantly longer stimulation and higher Gn doses than the control patients, but the IVF outcomes were comparable between the two groups. Furthermore, in our study, both GnRH-a and GnRH-ant regimes were evaluated while in previous studies, only a standard long protocol was discussed in the control group. In our study, HH patients were further divided into CHH and AHH subgroups, and the results indicated that although the etiology and basic treatment of HH subgroups were different, yet they have no difference in the outcomes of IVF treatment, which had not been reported in previous literature. CLBR has been suggested as a suitable way of reporting success of an IVF program which incorporates fresh as well as thawed frozen embryo transfer (17). From the patients’ perspectives, CLBR is more important since it better summarizes the chance of a live birth over an entire treatment period (18, 19). One highlight of our study is that the CLBR of all cycles was analyzed, and we found that the conservative and optimal CLBRs after second oocyte retrieval cycles could reach 58.02% and 68.45%, respectively. By the fourth cycle, they reached 60.49% and 74.19%, respectively. The above data suggested that it is worthwhile to try at least two cycles of oocyte retrieval for HH patients. A recent study of CLBRs based on multicenter reproductive clinical data from the general Chinese population indicated that by the fourth transfer cycle, the conservative and optimal estimates of CLBRs are 52.95% and 77.30% in women under the age of 30 (20). Another retrospective cohort study performed among 20,687 women undergoing IVF from 2007 to 2016 found that the conservative CLBRs of the first, the second, the third, and the fourth cycles are 50.74%, 60.11%, 62.88%, and 63.75%, respectively, while the corresponding optimal CLBRs are 50.74%, 65.87%, 73.51%, and 77.61% (21). The CLBR of HH patients we reported in the present study was similar to that of non-HH patients reported in the above literature.

Due to the lack of long-term hormonal stimulation, the size of the uterus in HH patients is small. Although the uterus size is promoted with HRT before ovulation induction, concerns still arose about whether pregnancy-related risk such as premature delivery is increased in HH patients. In our study, among the 7 PTBs, only 1 preterm infant delivered at 29 weeks of gestation, and the other 6 delivered at 36 weeks. All the preterm infants had satisfactory postpartum scores and were in good health. One patient of twin pregnancy suffered abortion because of premature rupture of membranes at 26 weeks of gestation after transferring two embryos in a fresh cycle, and then delivered at term after a single blastocyst transfer in an artificial cycle. One meta-analysis of cohort studies reported that PTB occurs in 10.1% IVF/ICSI and 5.5% spontaneously conceived pregnancies (22). Another prospective follow-up study conducted by Moini et al. reported that PTB rate of twins conceived by ART before 36 weeks could be as high as 51.30% (118/230) and 36% delivered at 32-36 weeks (23). In our study, the PTB rates of singletons and twin pregnancy in HH patients were 8.33% (3/36) and 30.77% (4/13), respectively, which were lower than the reported PTB rates, indicating that pregnancy-related risk was not increased in HH patients. To our knowledge, this study was the first to report the PTB rate in HH patients. It should be noted that due to the small size of twin live births, it is necessary to accumulate data for further confirmation. As twin pregnancies are at a higher risk, singleton pregnancy is strongly recommended for HH patients.

Our study firstly assessed the efficacy of different ovulation induction drugs in patients with HH. IVF-related parameters, such as hormone levels on HCG trigger day, Gn stimulation and dosage, No. of oocytes retrieved, fertilized embryos, transferable embryos, fertilization rate, and 2PN rate, were all comparable between the two groups, indicating that there was no difference in the effects between HMG alone and combination of rFSH and HMG. Compared with rFSH, HMG is much more cost-effective and could be selected as the preferred choice. Herein, ovulation induction with HMG alone is the most commonly used protocol in our center.

Anti-Mullerian hormone (AMH) is an established marker of reproductive potential used in recent years and it does not vary considerably with physiologic fluctuations in gonadotropin levels and remains reasonably stable throughout the menstrual cycle (24). Most of women with HH may have normal or high AMH levels (25, 26), while some other studies indicated that severe and/or prolonged deficiencies in GnRH and Gn production may lower AMH concentrations (27, 28).Whether AMH levels could be used to guide fertility treatment in women with HH still need further investigation. Due to the long span of our cases, AMH was not measured at that time. Therefore, our study was unable to analyze the relationship between AMH and ovarian response. In the further research, we will discuss the characteristics of AMH in HH patients and the application value in ovulation induction program in this population.

In conclusion, we found that the results of IVF-ET in HH patients were comparable to those in women with tubal factor of oviduct factor infertility and there was no difference in IVF outcomes among different subgroups of HH. For HH patients, HMG is cost-effective for ovulation and could be considered as the first choice. Encouragingly, the PTB rate was not increased and the CLBR of HH patients were satisfactory. Therefore, IVF-ET can be successfully employed in women with HH and patients can get good pregnancy outcomes.

## Data Availability Statement

The original contributions presented in the study are included in the article/**Supplementary Material**. Further inquiries can be directed to the corresponding author.

## Ethics Statement

The studies involving human participants were reviewed and approved by the Ethics Committee of Peking University Third Hospital. Written informed consent for participation was not required for this study in accordance with the national legislation and the institutional requirements.

## Author Contributions

YW, RL and JQ supervised the entire study and participated in the interpretation of the study data and revisions to the article. C-MZ collected the data and drafted the manuscript. RY and PL participated in the article drafting. HZ and L-XC conducted the statistical analysis. All authors contributed to the article and approved the submitted version.

## Funding

This work was supported by the National Natural Science Foundation of China (81601243, 81550022, and 81873833)

## Conflict of Interest

The authors declare that the research was conducted in the absence of any commercial or financial relationships that could be construed as a potential conflict of interest.

## Publisher’s Note

All claims expressed in this article are solely those of the authors and do not necessarily represent those of their affiliated organizations, or those of the publisher, the editors and the reviewers. Any product that may be evaluated in this article, or claim that may be made by its manufacturer, is not guaranteed or endorsed by the publisher.
