# Supplementary material for: The Reproductive Outcome of Women with Hypogonadotropic Hypogonadism in IVF
Source: Front Endocrinol (Lausanne). 2022 Jun 6;13:850126. doi: 10.3389/fendo.2022.850126 (PMC9208655; doi:10.3389/fendo.2022.850126)
Supplement: Supplementary file 1 [file Table_1.docx]

**SUPPLEMENTAL TABLE 1** Comparison of clinical characteristics and IVF related parameters between hypogonadotropic hypogonadism (HH) and the GnRHa-control group

| Variables | HH group (n=81) | GnRHa-control (n=59) | *P* |
| --- | --- | --- | --- |
| Age (y) | 30 (27, 32) | 30 (29, 31) | 0.953 |
| BMI (kg/m^2^) | 21.01 (19.53, 23.60) | 21.5 (18.95, 23.55) | 0.997 |
| Basal serum hormonal level |  |  |  |
| FSH (mIU/mL) | 1 (0.36, 2.88) | 6.49 (4.99, 8.4) | < 0.001 |
| LH (mIU/mL) | 0.3 (0.1, 0.78) | 3.39 (2.42, 4.5) | < 0.001 |
| E2 (pmol/L) | 82.2 (73.4, 119) | 168 (116, 217) | < 0.001 |
| A (nmol/L) | 4.54 (3.21, 6.06) | 6.3 (5.01, 8.37) | < 0.001 |
| PRL (ng/mL) | 5.65 (4.28, 8.68) | 12 (9.18, 20) | < 0.001 |
| Hormone levels on HCG day |  |  |  |
| E2 (pmol/L) | 6751 (4524.5, 12656) | 12463 (5804, 17294) | 0.012 |
| LH (mIU/mL) | 0.18 (0.11, 0.56) | 0.68 (0.47, 1.02) | < 0.001 |
| P (nmol/L) | 1.77 (1.42, 3.09) | 2.69 (1.66, 3.91) | 0.012 |
| Duration of stimulation (d) | 14 (13, 16) | 12 (11, 13) | < 0.001 |
| Total amount of Gn injected (IU) | 3487.5 (2850, 4500) | 2850 (2193.75, 3562.5) | < 0.001 |
| No. of oocytes retrieved | 11 (7, 14) | 13 (10, 19) | 0.005 |
| No. of fertilized embryos | 9 (5, 11) | 10 (7, 15) | 0.027 |
| No. of 2PN embryos | 6.5 (4, 9) | 9 (6, 11.5) | 0.010 |
| No. of non-2PN embryos | 1 (0, 2) | 1 (0, 3) | 0.983 |
| Fertilization rate (%) | 0.79 (0.64, 0.97) | 0.78 (0.63, 0.91) | 0.586 |
| 2PN rate (%) | 0.86 (0.72, 1) | 0.88 (0.75, 1) | 0.224 |
| No. of transferable  embryos | 3.5 (2, 8) | 4 (2, 9) | 0.547 |

BMI, body mass index; FSH, follicle stimulating hormone; LH, luteinizing hormone; E2, estradiol; A, androstenedione; PRL, prolactin; Gn, gonadotropin; PN, pronuclear. The data are expressed by the median (25% quantile, 75% quantile), and the comparison between the two groups is performed by Wilcox test.
