# Supplementary material for: The Reproductive Outcome of Women with Hypogonadotropic Hypogonadism in IVF
Source: Front Endocrinol (Lausanne). 2022 Jun 6;13:850126. doi: 10.3389/fendo.2022.850126 (PMC9208655; doi:10.3389/fendo.2022.850126)
Supplement: Supplementary file 2 [file Table_2.docx]

**SUPPLEMENTAL TABLE 2** Comparison of clinical characteristics and IVF related parameters between hypogonadotropic hypogonadism (HH) and the GnRH-ant-control group

| Variables | HH group (n=81) | GnRH-ant-control (n=53) | *P* |
| --- | --- | --- | --- |
| Age (y) | 30 (27, 32) | 31 (29, 32) | 0.42 |
| BMI (kg/m^2^) | 21.01 (19.53, 23.60) | 21.48 (19.95, 24) | 0.335 |
| Basal serum hormonal level |  |  |  |
| FSH (mIU/mL) | 1 (0.36, 2.88) | 6.81 (5.72, 7.94) | < 0.001 |
| LH (mIU/mL) | 0.3 (0.1, 0.78) | 3.33 (2.36, 4.95) | < 0.001 |
| E2 (pmol/L) | 82.2 (73.4, 119) | 155 (131, 227) | < 0.001 |
| A (nmol/L) | 4.54 (3.21, 6.06) | 5.8 (3.97, 8.5) | 0.04 |
| PRL (ng/mL) | 5.65 (4.28, 8.68) | 11.68 (8.54, 17.2) | < 0.001 |
| Hormone levels on HCG day |  |  |  |
| E2 (pmol/L) | 6751 (4524.5, 12656) | 7133 (5378, 11006) | 0.998 |
| LH (mIU/mL) | 0.18 (0.11, 0.56) | 2.16 (1.09, 3.2) | < 0.001 |
| P (nmol/L) | 1.77 (1.42, 3.09) | 2.28 (1.45, 3.43) | 0.306 |
| Duration of stimulation (d) | 14 (13, 16) | 10 (9, 11) | < 0.001 |
| Total amount of Gn injected (IU) | 3487.5 (2850, 4500) | 2100 (1550, 2700) | < 0.001 |
| No. of oocytes retrieved | 11 (7, 14) | 9 (6, 14) | 0.513 |
| No. of fertilized embryos | 9 (5, 11) | 6 (4, 10) | 0.214 |
| No. of 2PN embryos | 6.5 (4, 9) | 5 (3, 9) | 0.27 |
| No. of non-2PN embryos | 1 (0, 2) | 1 (0, 2) | 0.192 |
| Fertilization rate (%) | 0.79 (0.64, 0.97) | 0.75 (0.57, 0.83) | 0.148 |
| 2PN rate (%) | 0.86 (0.72, 1) | 0.83 (0.67, 1) | 0.901 |
| No. of transferable  embryos | 3.5 (2, 8) | 4 (2, 7) | 0.991 |

BMI, body mass index; FSH, follicle stimulating hormone; LH, luteinizing hormone; E2, estradiol; A, androstenedione; PRL, prolactin; Gn, gonadotropin; PN, pronuclear. The data are expressed by the median (25% quantile, 75% quantile), and the comparison between the two groups is performed by Wilcox test.
