# Supplementary material for: The Reproductive Outcome of Women with Hypogonadotropic Hypogonadism in IVF
Source: Front Endocrinol (Lausanne). 2022 Jun 6;13:850126. doi: 10.3389/fendo.2022.850126 (PMC9208655; doi:10.3389/fendo.2022.850126)
Supplement: Supplementary file 3 [file Table_3.docx]

**SUPPLEMENTAL TABLE 3** Comparison of clinical and cycle characteristics between congenital hypogonadotropic hypogonadism (CHH) and functional hypothalamic amenorrhea (FHA) groups

| Variables | CHH (n=41) | FHA (n=31) | *P* |
| --- | --- | --- | --- |
| Age (y) | 29 (27, 32) | 31 (28.5, 34) | 0.070 |
| BMI (kg/m^2^) | 21.48 (19.48, 24.24) | 20.94 (19.38, 23.74) | 0.562 |
| Basal serum hormonal level |  |  |  |
| FSH (mIU/mL) | 0.83 (0.28, 2.98) | 1 (0.4, 2.21) | 0.918 |
| LH (mIU/mL) | 0.27 (0.1, 1.02) | 0.39 (0.1, 0.7) | 0.770 |
| E2 (pmol/L) | 91.4 (73.4, 139) | 73.4 (73.4, 108) | 0.152 |
| A (nmol/L) | 4.7 (3.77, 6.2) | 4.61 (3.22, 6.39) | \| 0.814 \| \| --- \| |
| PRL (ng/mL) | 5.6 (4.58, 6.9) | 5.3 (3.13, 8.25) | 0.530 |
| Hormone levels on HCG day |  |  |  |
| E2 (pmol/L) | 6575 (4302, 10984) | 7026 (5914, 13210) | 0.463 |
| LH (mIU/mL) | 0.18 (0.11, 0.58) | 0.19 (0.11, 0.45) | 0.723 |
| P (nmol/L) | 1.69 (1.34, 3.4) | 2.03 (1.62, 3.05) | 0.640 |
| Duration of stimulation (d) | 14 (13, 17) | 14 (13, 15) | 0.649 |
| Total amount of  Gn injected (IU) | 3537.5 (3018.75, 4631.25) | 3375 (2325, 4237.5) | 0.242 |
| No. of oocytes retrieved | 11 (7, 12) | 12 (9, 16) | 0.057 |
| No. of fertilized embryos | 8 (5, 9) | 9 (7, 12.5) | 0.082 |
| No. of 2PN embryos | 6.5 (3,9) | 7 (6,10) | 0.190 |
| No. of non-2PN embryos | 1 (0, 2) | 2 (0.5, 2) | 0.361 |
| Fertilization rate | 0.77 (0.63, 0.94) | 0.78 (0.65, 0.92) | 0.972 |
| 2PN rate | 0.85 (0.67, 1) | 0.85 (0.75, 0.98) | 0.550 |
| No. of transferable  embryos | 3 (2, 8.25) | 4 (2, 8.5) | 0.242 |

BMI, body mass index; FSH, follicle stimulating hormone; LH, luteinizing hormone; E2, estradiol; A, androstenedione; PRL, prolactin; Gn, gonadotropin; PN, pronuclear. The data are expressed by the median (25% quantile, 75% quantile), and the comparison between the two groups is performed by Wilcox test.
